# Wetland Expansion Reduces CO_2_ ‐Equivalent Emissions and Strengthens the Congo Basin's Role as a Net Carbon Sink

**DOI:** 10.1111/gcb.70746

**Published:** 2026-02-12

**Authors:** Aidan Byrne, Jake Williams, Nathalie Pettorelli

**Affiliations:** ^1^ Institute of Zoology Zoological Society of London London UK; ^2^ University College London London UK

**Keywords:** carbon storage, climate change, greenhouse gas, hydrology, peatlands, remote sensing, swamp forest

## Abstract

Wetlands are the largest natural source of methane, yet their desiccation releases substantial amounts of carbon dioxide. Changing wetland emissions provide the greatest source of uncertainty in global emissions estimates due to limited data for key tropical carbon sources and sinks, including the Congo Basin. Here we quantified changing swamp forest hydrology, forest productivity and greenhouse gas emissions between 2007 and 2024 using satellite Earth observation and emissions datasets. We show that swamp forests expanded from 195,345 km^2^ to 222,467 km^2^ between 2007 and 2024, demonstrating a reversal of previously reported long‐term drying trends. The observed wetting trend increased productivity in both swamp and *terra firme* forests. Despite increasing methane emissions, wetland expansion reduced CO_2_‐equivalent emissions by 2 (95% CI; −2.94 to −1.12) million tonnes per year since 2007, highlighting the region's increasing role as a net carbon sink and its significance for global carbon budgets.

## Graphical abstract

Congo Basin peat swamp forests are vital carbon sinks, yet climate‐driven shifts in hydrology and wetland greenhouse gas fluxes have not been assessed. Here we quantified changes in swamp forest extents, water table levels, forest productivity and wetland greenhouse gas fluxes between 2007 and 2024. Contrary to previously reported long‐term drying trends, we found that swamp forests expanded by 14%. Despite increasing methane emissions, wetland expansion reduced CO_2_‐equivalent emissions by 2 million tonnes per year since 2007. Our findings highlight the Congo Basin's increasing role as a net carbon sink and its significance for global carbon budgets.

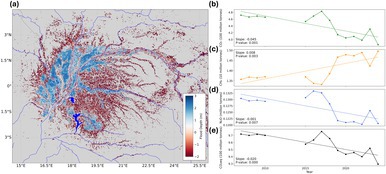

## 1 Introduction

Wetlands store over one‐third of global soil organic carbon, yet these ecosystems are the largest natural source of methane (CH_4_) emissions (Zou et al. 2022). Globally, 21% of wetland area has been lost since 1700 (Fluet‐Chouinard et al. 2023), exposing these dense soil carbon stores to decomposition and influencing emissions of carbon dioxide (CO_2_), CH_4_ and nitrous oxide (N_2_O) (Zou et al. 2022). Despite their central role in the global carbon cycle, wetland processes have only recently been integrated into Earth System Models (ESMs) (Forbrich et al. 2024; Sjögersten et al. 2014). There, wetland CH_4_ emissions are both the largest natural source term and carry the greatest proportional uncertainty. Addressing uncertainties such as this one is a key research development identified by the International Panel on Climate Change (IPCC) Seventh Assessment Report (IPCC 2023). Model uncertainties have been attributed to inaccurate assessments of wetland extent (Zou et al. 2022), poor representation of tropical wetlands in global emissions datasets (Borges et al. 2015; Forbrich et al. 2024; Ribeiro et al. 2021), the aggregation of different wetland ecosystems into a single wetland class (Forbrich et al. 2024) and the omission of methane‐climate feedbacks (Zhang et al. 2023). Regional temperature and rainfall patterns largely determine wetland hydrology and, consequently, greenhouse gas (GHG) fluxes (Zou et al. 2022); however, the impacts of climate change on remote tropical wetlands, particularly in Africa, remain poorly understood. The accurate quantification of tropical wetland dynamics in response to recent climatic changes is essential for improving GHG emissions estimates in ESMs.

The world's two largest river basins, the Amazon and the Congo, contain extensive swamp forests and peatlands (Dargie et al. 2017; Pärn et al. 2023), storing large quantities of CO_2_ below ground while emitting substantial amounts of CH_4_ and N_2_O (Barthel et al. 2022; Pärn et al. 2023). The significance of the Congo Basin's carbon stocks has recently come to light, due to the identification and mapping of the world's largest tropical peatland complex, the Cuvette Centrale (Crezee et al. 2022; Dargie et al. 2017). These peat swamps store an estimated 29 petagrams of carbon below ground, equivalent to the aboveground carbon stocks of all Congo Basin tropical forests (Crezee et al. 2022). Despite its importance, the region's hydrology remains poorly understood (Dargie et al. 2019) and consistent flood mapping is lacking (Papa et al. 2023). The wetlands of the Congo Basin are estimated to emit similar or even greater amounts of carbon per unit area than those of the Amazon; however, the Congo remains massively understudied, largely due to the lack of climate and hydrological observations for the region (Alsdorf et al. 2016). Wetland emissions respond nonlinearly to hydrological shifts; peatland desiccation promotes aerobic decomposition and the release of CO_2_, whereas flood inundation enhances the anaerobic conditions that drive CH_4_ emissions (Zou et al. 2022). Thus, the scarcity of hydrological data for the Congo Basin poses major challenges for accurate emissions estimates.

The Congo Basin experienced a long‐term drying trend between 1988 and 2013, marked by a widespread increase in the length of the boreal summer dry season (Jiang et al. 2019), contributing to a widespread decline in rainforest greenness (photosynthetic capacity) between 2000 and 2012 (Zhou et al. 2014). Whether this long‐term drying trend led to a decline in the region's wetlands, exposing the soil carbon stores and amplifying CO_2_ emissions, has not been assessed. More recent rain gauge and satellite data, however, identified a wetter period between 2016 to 2020 (Nicholson et al. 2022), and no continued decline in rainforest leaf area was detected when Moderate Resolution Imaging Spectroradiometer (MODIS) data up to 2019 were included (Sun et al. 2022). The impacts of these regional climatic shifts on wetland dynamics and GHG emissions must be quantified to determine the stability of the Congo Basin's role as a net carbon sink (Garcin et al. 2022). In the present study, we integrate high‐resolution Synthetic Aperture Radar (SAR), hydrological modelling, MODIS vegetation indices and wetland emissions datasets to test three main predictions: (1) long‐term drying trends reduced Congo swamp forest extent, (2) declining flood inundation suppressed wetland forest productivity, (3) swamp forest loss and peatland desiccation drove increasing CO_2_ emissions and decreasing CH_4_ emissions. We evaluate our findings against regional climatic and hydrological data, a low spatial resolution global wetland dataset representative of the wetland data used in ESMs, as well as previous investigation done over the Congo and Amazon basins. Finally, we discuss the implications of our findings for improving wetland dynamics and GHG emissions in ESMs and global carbon budgets.

## 2 Results

### 2.1 Swamp Forest Extent

**FIGURE 1:**
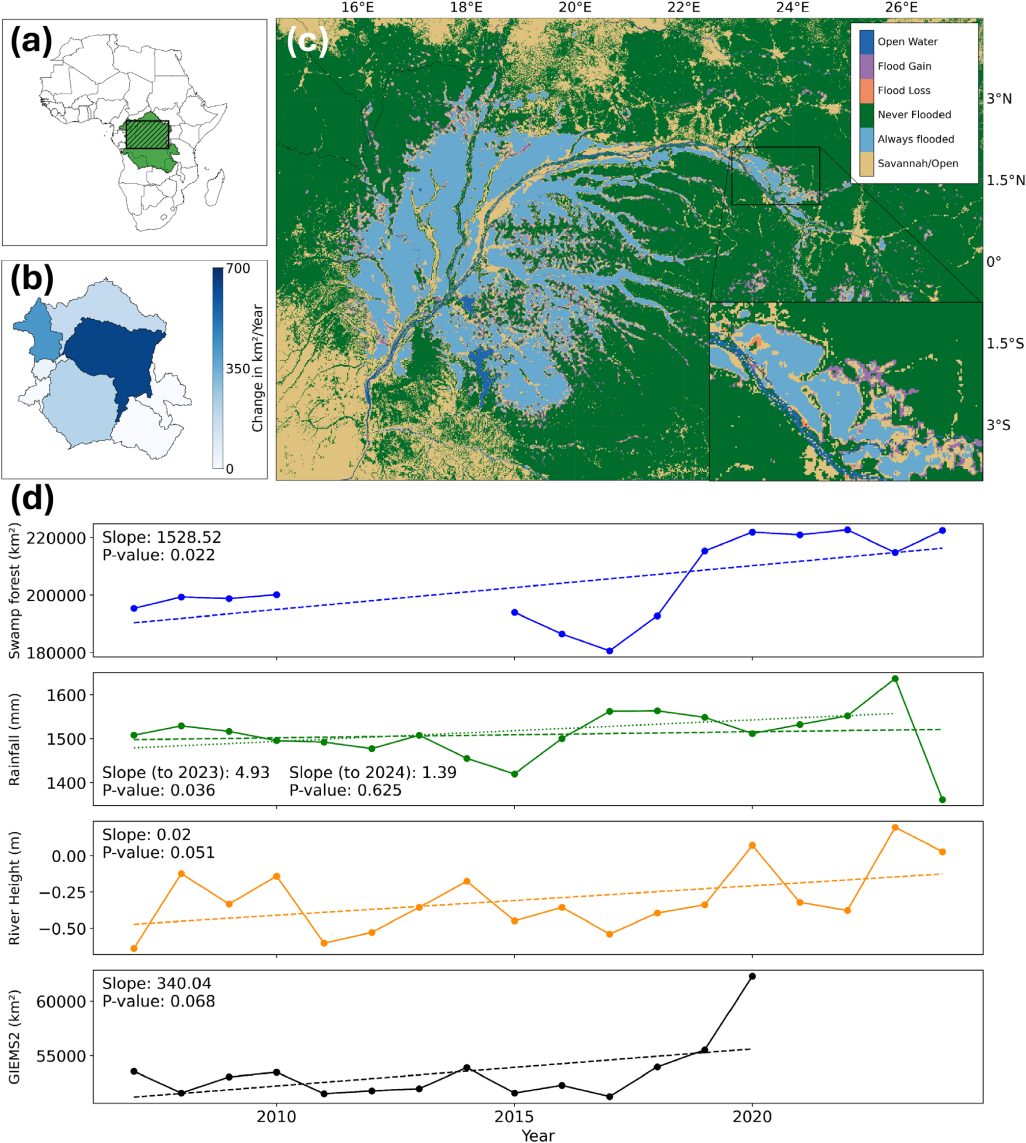
Spatial and temporal variability in swamp forest extent from 2007 to 2024. (a) The location of the Congo Basin (green) within the African continent. The shaded rectangle represents the extent of box C. Map lines delineate study areas and do not necessarily depict accepted national boundaries. (b) Temporal trends in swamp forest extent for the nine subbasins within the Congo Basin (see Table S2 for full results). (c) Landcover map visualising the extent of permanently flooded swamp forest, *terra firme* (non‐flooded) forest, as well as regions that experienced flood loss and flood gain between 2007 and 2024. The inset shows a close‐up of swamp forest loss and gain around an eastern portion of the Congo mainstem. (d) Swamp forest extent (blue), Climate Hazards Group InfraRed Precipitation with Station data (CHIRPS) rainfall (green), mean river height anomaly compared to a Jason‐2 or Jason‐3 baseline for 52 virtual stations across the Congo Basin (orange) and wetland extent estimates from the GIEMS‐2 dataset from 2007 to 2020 (black). Data gaps for swamp forest extents are due to no overlap between the ALOS PALSAR and PALSAR‐2 satellites (2011 to 2014). Rainfall trends are visualised up to 2024 (dashed line) and 2023 (dotted line) to show the effect of the anomalously dry year in 2024.

Contrary to our first prediction, swamp forest extent significantly increased by 1528 km^2^ (95% confidence interval [CI]; 265–2791 km^2^) per year, from 195,345 km^2^ (95% CI; 184,742–205,947 km^2^) in 2007 to 222,467 km^2^ (95% CI; 210,393–234,542 km^2^) in 2024 (Figure 1d and Table S1). Capitalising on the double‐bounce mechanism of PALSAR L‐band SAR backscatter in inundated forests (Georgiou et al. 2023) and an extensive dataset of GPS ground‐truth coordinates for model training and validation (Crezee et al. 2022), we classified annual swamp forest extent between 2007 and 2024 across the Congo Basin. Flooding extent increased the most in the Middle Congo (646 km^2^ per year), the subbasin containing the eastern section of the Cuvette Centrale and the largest portion of the Congo River mainstem (Figure 1a–c, Table S2). Our swamp forest extent estimates were 356% larger than the wetland areas derived from the Global Inundation Extent from Multi‐Satellites (GIEMS‐2) dataset (based on 2020 annual extents; Figure 1d), demonstrating the underestimation of tropical swamp forests in low spatial resolution global grid cell water fraction datasets typically used in ESMs (Forbrich et al. 2024). The observed swamp forest expansion coincided with a significant increase in basin‐wide annual average rainfall between 2007 and 2023 (Slope = 4.93, *p*‐value = 0.036), followed by an anomalously dry year in 2024 (Figure 1d). Further, annual river level heights averaged across 52 stations throughout the basin increased over the study period (Slope = 0.02, *p*‐value = 0.051; Figure 1d), providing further evidence of a wetting trend. A distinct shift from a drying trend to a wetting trend was observed in all hydrological metrics between 2015 and 2019 (Figure S1). Validation of swamp forest extents and hydrological trends is discussed further in the Supporting Information.

### 2.2 Forest Productivity

**FIGURE 2:**
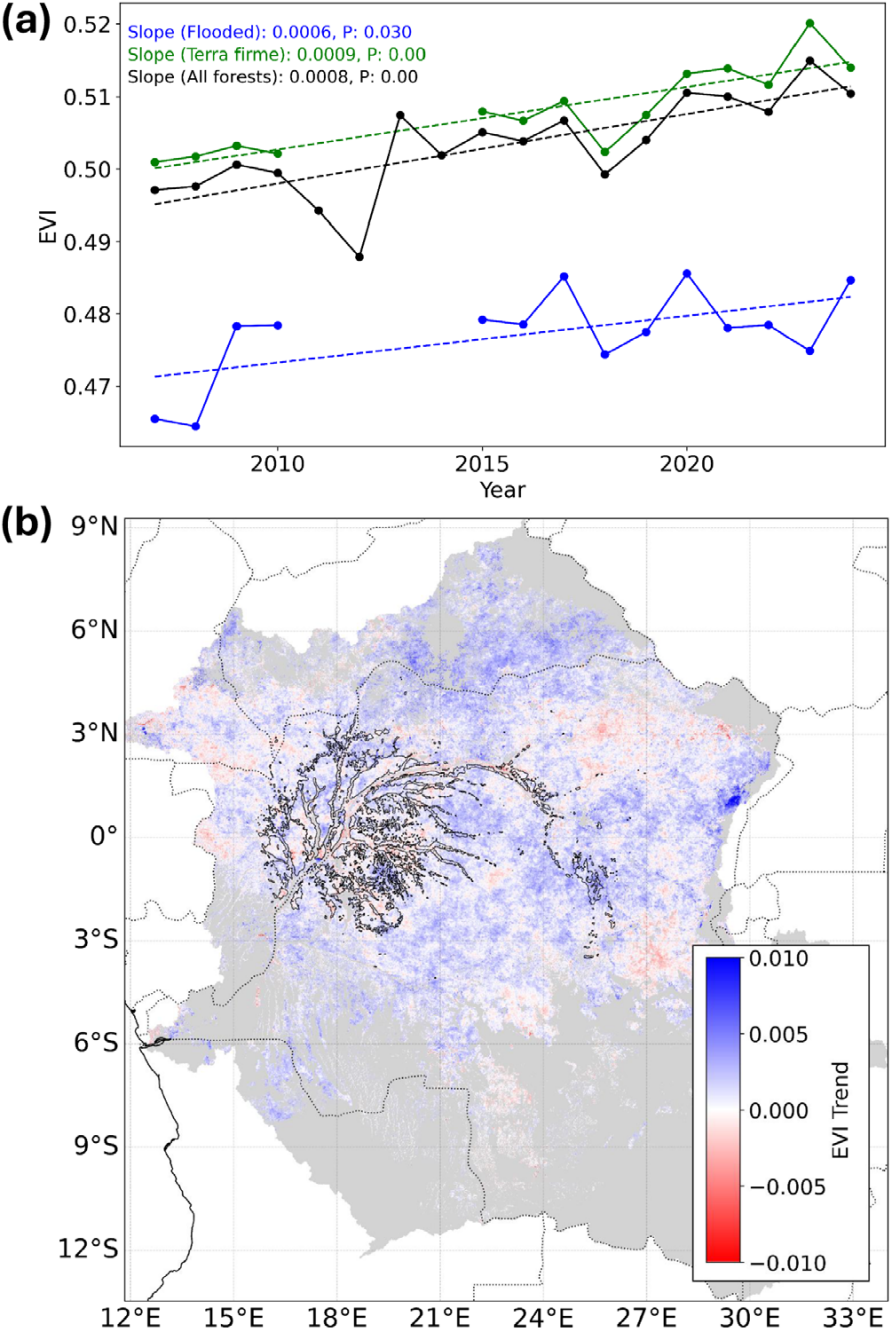
Increasing productivity (EVI) in all forests, swamp forests and *terra firme* forests for the April–May–June period between 2007 and 2024. (a) Mean annual EVI for all tropical forests (black), permanently flooded swamp forests (blue) and *terra firme* forests (green). Dashed lines represent trend lines. (b) Pixel‐wise trends in EVI for all tropical forests within the Congo Basin. Black polygons delineate the extent of permanently flooded swamp forests over the study period.

Vegetation biomass production determines the carbon storage potential of peatlands and influences methane production in wetlands through leaf litter and the supply of labile organic matter (Forbrich et al. 2024). If the previously reported forest browning trend in the Congo Basin has persisted (Zhou et al. 2014), the decline in biomass production should reduce the carbon storage potential of the Congo's peat‐forming swamp forests. For consistency with previous research (Zhou et al. 2014), we quantified MODIS Enhanced Vegetation Index (EVI), a measure of photosynthetically active vegetation and a proxy for productivity, for the April–May–June period (the most cloud free period) between 2007 and 2024 for the Congo Basin's tropical forests. We identified a reversal in the reported browning trend, with mean EVI for all Congo Basin forests significantly increasing over the study period (Slope = 0.001, *p*‐value < 0.001; Figure 2a). Pixel‐wise analyses identified that 10.42% of pixels had a significant increasing trend in EVI (*p*‐value < 0.05), whereas just 1.33% of pixels had a significant decreasing trend (*p*‐value < 0.05; Figure 2b). We did observe the reported browning trend from 2000 to 2012 in our dataset; however, EVI values rebounded in 2013 and continued along an increasing trend (Figure 2a). Swamp forests were significantly less productive than *terra firme* forests (*t*‐statistic = −20.77, *p*‐value < 0.001). Nevertheless, both swamp forests (slope = 0.0006, *p*‐value = 0.03) and *terra firme* forests (slope = 0.0009, *p*‐value < 0.001) increased in productivity over the study period.

### 2.3 Swamp Forest GHG Emissions

**FIGURE 3:**
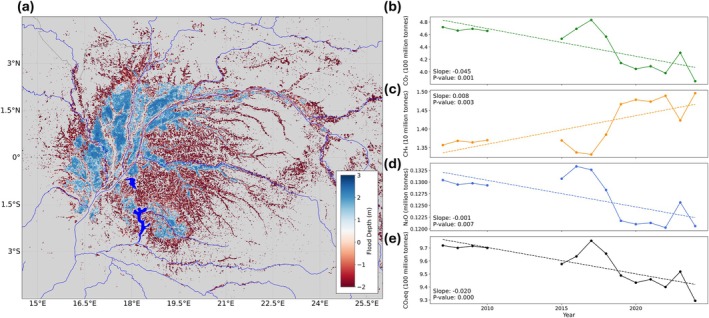
Swamp forest water table levels and annual GHG emissions from 2007 to 2024. (a) Swamp forest water table levels for the year 2020. Water table levels were estimated for each year relative to the maximum swamp forest extent observed over the study period. Dark blue lines and polygons represent lakes and rivers. Total annual wetland emissions derived from swamp forest extents and water table levels for (b) carbon dioxide (CO_2_) in 100 million tonnes, (c) methane (CH_4_) in 10 million tonnes, (d) nitrous oxide (N_2_O) in million tonnes and (e) CO_2_‐equivalent in 100 million tonnes (100‐year horizon).

Wetland methane emissions increase with both spatial extent and flood depth, with the largest emissions coming from waters deeper than 40 cm, whereas CO_2_ emissions peak as water table levels recede to below ground surface level (Zou et al. 2022). We accounted for this by predicting swamp forest water table levels, using the annual flood extent maps and the inverse of the Height Above Nearest Drainage (HAND) model relative to the maximum flood depth for each year (Figure 3a). We then combined these water table level extents with a global tropical wetland GHG emissions database (Zou et al. 2022; Table S3). The expansion of swamp forests equated to an increase of 80,000 (95% CI; 30,000–120,000) tonnes per year of CH_4_ emissions, from 13.57 (95% CI; 8.02–19.12) million tonnes in 2007 to 14.96 (95% CI; 8.88–21.04) million tonnes in 2024 (Figure 3c and Table S4). Previous studies using ensemble models from the WetCHARTs database estimate a range of CH_4_ emissions for the Congo Basin, from 2 to 21 million tonnes per year (Bloom et al. 2017), with our results falling towards the centre of this range. Conversely, CO_2_ emissions decreased by 4.5 (95% CI; −6.7 to −2.2) million tonnes per year, whereas N_2_O emissions declined by 570 (95% CI; −950 to −190) tonnes per year, as forests with below‐ground water table levels became inundated (Figure 3b,d). Despite the increase in methane, CO_2_‐equivalent emissions, a measure of global warming potential, from the Congo swamp forests declined by 2 (95% CI; −2.94 to −1.12) million tonnes per year between 2007 and 2024 when considered over a 100‐year horizon (Figure 3e). Declines were greatest between 2017 and 2024 (−4.3 million tonnes per year), coinciding with the rapid expansion of swamp forests (Figure S1h).

## 3 Discussion

Although the Congo Basin is considered the most pristine large basin left on Earth, it is particularly vulnerable to climatic change. It receives considerably less rainfall than the Amazon (1.53 m/year compared to 2.13 m/year; Alsdorf et al. 2016), and the Cuvette Centrale's vast peat carbon stocks lie close to a climatically driven drought threshold, below which the region could shift from a carbon sink to a carbon source (Garcin et al. 2022; Young et al. 2023). Unlike the Amazon's fluvially flooded wetlands, Congo swamp forests are largely rainfed (Alsdorf et al. 2016; Georgiou et al. 2023), meaning inundation of the Cuvette Centrale is more directly governed by regional rainfall, surface temperatures and evapotranspiration than by upstream river discharge. As a result, this vital carbon sink is highly sensitive to regional climatic fluctuations. The recent wetting trend reported for 2016 to 2020, further supported by our results (Figure S1), was likely driven by an increase in convective available potential energy and in total column water vapour related to warming ocean and land surface temperatures (Nicholson et al. 2022). However, it remains uncertain whether this wetting trend will persist or if the long‐term drying trend will prevail (Jiang et al. 2019), due to limited climate observations and inconsistent rainfall projections for the region (Alsdorf et al. 2016). Our unexpected result of swamp forest expansion demonstrates this uncertainty, highlights the hydrological sensitivity of the Congo Basin's swamp forests to short‐term climatic variability and provides a useful proxy for regional climatic changes in the absence of accurate climate datasets.

Here, we reveal a reversal of the previously reported browning trend for Congo forests (Zhou et al. 2014), with increased photosynthetic activity observed in both swamp and *terra firme* forests. On average, swamp forests were less productive than *terra firme* forests (Figure 2a). These results are consistent with observations of lower productivity in Peruvian peat swamps compared to *terra firme* forests, attributed to the anaerobic soil conditions of swamp forests and its effects on photosynthesis and respiration efficiency (Dargie et al. 2024). Regardless, the observed greening of both forest ecosystems provides further evidence of a wetting trend in the basin, as tropical forest productivity increases with enhanced water availability (Sun et al. 2022). These results suggest increasing vegetation biomass and leaf litter production, which likely enhanced the carbon sequestration potential of the region's peat‐forming swamp forests (Sjögersten et al. 2014), albeit with increasing capacity for methane production (Forbrich et al. 2024). Increasing wetland vegetation biomass has also been linked to greater downstream riverine CO_2_ and CH_4_ emissions through organic carbon inputs (Borges et al. 2015). Enhanced hydrological connectivity under wetter conditions also increases the likelihood that the additional organic carbon inputs will be exported downstream (Norouzi et al. 2024), highlighting the wider implications for inland water emissions. Our emissions estimates did not account for changes in organic matter inputs, however, and the influence of increasing forest productivity on swamp forest GHG fluxes requires in situ experimental studies. Increasing wetness may also trigger ecological trade‐offs that are not captured by our remote‐sensing metrics. Prolonged inundation can reduce species richness and shift community composition toward more flood‐tolerant but lower‐biomass taxa, reducing carbon stocks in previously dry forests (Hawes et al. 2012; Kompanyi et al. 2025). Therefore, hydrological change drives both carbon gains and losses within these ecosystems and ecological feedbacks must be considered for improved estimates of the Congo Basin carbon budget.

Swamp forest expansion since 2007 reduced the total CO_2_‐equivalent emissions from the Congo Basin, increasing the region's role as a net carbon sink (Garcin et al. 2022). This reduction was driven by rising water table levels stabilising the region's vital soil organic carbon stocks through reduced aerobic decomposition, limiting CO_2_ and N_2_O emissions (Figure 3). However, water table levels alone do not fully control wetland GHG dynamics; fluxes are further regulated by plant functional traits such as aerenchyma‐mediated methane transport, soil organic carbon content, nutrient availability and microbial community composition (Cao et al. 2024; Joabsson et al. 1999). Though these factors were not explicitly accounted for in our modelling framework, the uncertainty they could add into our flux estimates was largely accounted for by using GHG flux averages (with variability) across tropical wetland types that capture a range of environmental conditions (Zou et al. 2022). While this database includes a small number of sites in Central Africa, the use of global tropical averages was necessary to capture the range of potential GHG flux values (hence the wide confidence intervals) in the absence of sufficient region‐specific data. The main source of uncertainty for our flux estimates was that below‐ground water table levels were only estimated within the maximum flooding extent observed over the study period; water table levels conducive for increased CO_2_ and N_2_O production would extend beyond these boundaries, meaning these emissions were likely underestimated in recent years as water levels rose. In contrast to the reduction in CO_2_‐equivalent emissions, CH_4_ emissions significantly increased with the expansion of swamp forests (Figure 3). The Congo Basin is already a net source of CH_4_ on average, despite 93% of its area (largely *terra firme* forests) being identified as a CH_4_ sink (Barthel et al. 2022), highlighting the increasing source strength of the swamp forests. Our CO_2_‐equivalent emissions for CH_4_ assume a global warming potential of 34 (relative to CO_2_) over a 100‐year horizon (Zou et al. 2022), consistent with IPCC reporting conventions (IPCC 2013). However, over a 20‐year horizon, methane's global warming potential is 82.5 times stronger than CO_2_ (IPCC 2021). Using the 20‐year horizon instead amplifies methane's contribution to CO_2_‐equivalent emissions, shifting toward a stronger near‐term radiative source and mitigating the reduced climate forcing driven by declining CO_2_ fluxes (Figure S2). Methane also acts as a precursor to tropospheric ozone (O_3_), adversely affecting plant production, reducing carbon absorption in terrestrial ecosystems and impacting human health (Mar et al. 2022). It is, therefore, essential to look beyond long‐term CO_2_‐equivalent emissions and assess shorter‐term GHG fluxes driven by changing wetland dynamics.

In this study, machine‐learning geospatial approaches were combined with a global tropical wetland GHG flux dataset to address the sparsity of hydrological and GHG flux data for Congo Basin swamp forests. To provide further confidence in our results, we performed the following actions: (1) trained and validated the swamp forest classification using the most comprehensive ground‐based GPS dataset available for the region (Crezee et al. 2022); (2) validated increasing swamp forest extent trends with observations of increasing basin‐wide annual average rainfall, river level heights and wetland extents from the GIEMS‐2 dataset (Figure 1d); (3) predicted water table levels for a more refined application of GHG flux measurements to swamp forest extents; (4) compared annual swamp forest extents (Supporting Information) and emissions (Bloom et al. 2017) to previous estimates; and (5) provided clear data on prediction uncertainties for swamp forest extents and annual GHG fluxes (Table S1, Table S4). Overall, this combination of validation approaches provides high confidence in the observed trends.

Here we show that current low spatial resolution global wetland datasets used in ESMs, such as grid cell water fraction or surface water storage (Forbrich et al. 2024; Lawrence et al. 2019), substantially underestimate the extent of swamp forests in the Congo Basin. This translates to a considerable underestimation of CH_4_ emissions from tropical wetlands. Despite this underestimation of methane emissions in previous datasets, we observed significant swamp forest expansion that demonstrates a reversal of the previously reported long‐term drying trend in the Congo Basin (Jiang et al. 2019), leading to a decline in CO_2_ and N_2_O emissions and the strengthening of the region's role as a net carbon sink. By applying high‐resolution, ecosystem‐specific methods, not only do we capture the true spatial extent of these wetlands, but also distinguish swamp forests from other wetland types and account for variation in water table levels. This information is vital for producing ecosystem‐specific GHG emissions estimates (Forbrich et al. 2024). Upscaling these methods would improve wetland representation in remote tropical regions and reduce the uncertainties in bottom‐up GHG modelling approaches (Sjögersten et al. 2014). Incorporating these methodologies into ESMs is essential for addressing the uncertainties in global wetland emissions source terms and for contributing to more accurate assessments of global carbon budgets under a changing climate.

## 4 Materials and Methods

### 4.1 Swamp Forest Extent

Swamp forests were mapped between 2007 and 2024 using L‐band microwave SAR imagery from the ALOS PALSAR and PALSAR‐2 satellites (25 m pixel size). Forested wetlands have a higher backscatter signal than *terra firme* forests due to the double‐bounce mechanism, where the 24 cm SAR wavelengths penetrate the canopy, reflect off the water's surface and scatter from nearby vegetation (Georgiou et al. 2023). ALOS PALSAR and PALSAR‐2 data were obtained from the Japan Aerospace Exploration Agency (JAXA) and accessed through Google Earth Engine (Gorelick et al. 2017). ALOS PALSAR annual mosaics were acquired for 2007 to 2010, and PALSAR‐2 annual mosaics were derived from PALSAR‐2 ScanSAR Level 2.2 imagery for 2015 to 2024. Low quality layover and shadowing pixels were removed using the image data quality flags; PALSAR‐2 pixel values were corrected for incidence angle for consistency with ALOS PALSAR (Georgiou et al. 2023), images were speckle filtered using a 3 by 3 squared‐kernel filter (Grimaldi et al. 2020) and digital numbers were converted to gamma naught values in decibels (dB). Partial data gaps in the 2008 ALOS PALSAR annual mosaic image (affecting two overpass paths in the Cuvette Centrale) were filled using the 2007 pixel values to improve the consistency of wetland extent estimates.

Tropical forests with flooding potential were then delineated by removing non‐forest pixels (Turubanova et al. 2018), high ground (Yamazaki et al. 2017) and areas with a Height Above Nearest Drainage (HAND) value greater than 20 m (Jensen et al. 2018). The HAND model is a terrain‐derived hydrological metric providing a measure of how high a pixel is above the nearest drainage channel, with values of 0 indicating rivers, streams and watercourses. For the remaining flood‐potential forest pixels, a random forest (RF) classification was trained using ground‐truth GPS coordinates for permanently flooded peat‐forming swamp forests and *terra firme* forests (*n* = 1087) (Crezee et al. 2022). Here, permanently flooded swamp forest refers to non‐seasonal pixels with flood inundation throughout a calendar year. The classification algorithm was trained (500 trees) on the 2019 annual PALSAR‐2 image to coincide with the recent GPS field data sampling campaign. The training dataset consisted of 70% of the ground‐truth datapoints, with 30% reserved for validation and accuracy assessment. Model inputs were PALSAR HH, HV and HV/HH polarisations, as well as HAND values, facilitating the identification of all swamp forests across the basin through their L‐band backscatter values and flooding potential. The feature space of HH, HV, HV/HH ratio and HAND values showed clear bimodality in backscatter distributions between permanently flooded and *terra firme* forests. Although seasonally flooded forests represent a significant contribution to wetland extent dynamics, they occupy an intermediate and more variable region of this feature space and are less likely to be associated with peat‐forming swamps. Due to the absence of available georeferenced samples for seasonally flooded forests and limited PALSAR revisit frequency between 2007 and 2010, we trained our classification on only permanently flooded and *terra firme* forests. The RF algorithm performed well with an overall accuracy of 79% (Swamp forests: User accuracy (UA) = 0.80, Producer accuracy (PA) = 0.73, *Terra firme*: UA = 0.78, PA = 0.84). Swamp forest extents were then mapped for each year by applying the trained RF classifier to each annual PALSAR image.

To assess temporal changes in swamp forest extent, we quantified the trend in annual swamp forest area between 2007 and 2024 using a linear regression model. No basin‐wide L‐band acquisitions were available from PALSAR imagery between 2011 and 2014, necessitating analysis of the full continuous record between 2007 and 2024. This data gap adds uncertainty to the trend analysis; however, there were sufficient annual data points either side of this window to provide confidence in the observed trend. Regression models were also developed independently for the nine subbasins within the Congo Basin to assess spatial changes in flooding extent using HydroSHEDS Basins Level 4 polygons (Linke et al. 2019). Further, pixel‐wise estimates of flood loss and flood gain were quantified by comparing flood presence in the ALOS PALSAR 2007 to 2010 period (coinciding with previously published estimates of wetland extent; Crezee et al. 2022; Dargie et al. 2017) with the PALSAR‐2 images from 2015 to 2024 (Figure 1c).

Temporal trends in swamp forest extent were validated by comparison with basin‐wide annual average rainfall from the Climate Hazards Group InfraRed Precipitation with Station data (CHIRPS) (Funk et al. 2015) dataset and satellite altimeter‐derived annual river level height anomalies for 52 virtual river stations across the Congo Basin (https://blueice.gsfc.nasa.gov/gwm). Spatial extents were further validated by comparison to the Global Inundation Extent from Multi‐Satellites (GIEMS‐2) dataset (Prigent et al. 2020) (approximately 25 km pixel size at the equator), representative of the low spatial resolution global wetland datasets used in ESMs. Piecewise regression models were also developed to identify consistent climatological shifts across the hydrological validation metrics and the swamp forest extent estimates (Figure S1). Here we used one breakpoint (two segments) to further address the uncertainty caused by the PALSAR and PALSAR‐2 imagery data gap, enabling direct validation of the trends in the 2007 to 2010 and 2015 to 2024 periods. Finally, our swamp forest extent results were also compared to previously published estimates of peat‐forming swamp forests and total wetland extent (Bwangoy et al. 2010; Crezee et al. 2022; Dargie et al. 2017), as well as subregional extents (Lee et al. 2011; Supporting Information).

### 4.2 Forest Productivity

We used the MODIS monthly global vegetation indices 1 km product (Didan 2021) to quantify forest EVI between 2007 and 2024. This study extends the Zhou et al. (2014) analysis, from 2000 to 2012, through to 2024. EVI values were obtained for the April–May–June period (lowest cloud coverage) and pixels with less than 80% data availability across the timeseries were removed for consistency across years. Annual mean EVI values for each pixel were obtained from the April–May–June images and non‐forested pixels were masked from further analyses (Turubanova et al. 2018). To assess forest productivity in permanently flooded swamp forests and *terra firme* forests, mean EVI was determined for pixels identified as always flooded (14/14 flood frequency) and never flooded (0/14 flood frequency). Changes in mean annual EVI were then quantified for all forests, swamp forests and *terra firme* forests using linear regression models. A paired *t*‐test was used to assess if the productivity of swamp forests was significantly lower than *terra firme* forests over the study period. The spatial distribution of EVI trends was further assessed using pixel‐wise trend analyses to determine if the previously reported widespread browning trend persisted beyond 2012 (Zhou et al. 2014).

### 4.3 Swamp Forest GHG Emissions

Wetland emissions of CO_2_, CH_4_ and N_2_O are largely determined by flood extent and water table level (WTL), with deeper waters emitting larger quantities of CH_4_ and shallower WTLs emitting increased CO_2_ in tropical wetlands (Zou et al. 2022). To estimate flood depths and WTLs in the absence of ground‐based water table data for Congo Basin swamp forests, the annual swamp forest maps were combined with the HAND model. Flood depths were determined relative to the maximum HAND value for each annual flood extent classification; the 90th percentile value (Retallack et al. 2024) was used to mitigate the effect of isolated flood patches at higher HAND values that were not representative of the wider flood depths. Each flooded pixel's HAND value was inverted relative to this maximum threshold, providing an estimate of flood depth for each annual classification (Nobre et al. 2016). To predict below‐ground WTLs, we first delineated the maximum flood extent across all years. For each year, we identified the minimum HAND value (10th percentile) from the non‐flooded pixels within this maximum flood extent. Below‐ground WTLs were then estimated from the non‐flooded pixel's HAND values relative to this minimum HAND threshold.

Wetland emissions estimates of CO_2_, CH_4_, N_2_O and CO_2_‐equivalent were obtained from a synthesis of 504 global GHG net flux experimental studies (Zou et al. 2022). Wetland GHG flux measurements for the Congo Basin are scarce (Barthel et al. 2022). Thus, we used the emissions estimates for tropical wetlands and the WTL aggregations defined by Zou et al. (2022) (Table S3). This dataset incorporates GHG flux measurements from tropical swamp forests, as well as other tropical wetland types and facilitates the inclusion of variable emissions estimates for different water table levels. Moreover, confidence intervals are provided to encompass the range of flux values from different tropical wetlands, providing further confidence in our emissions estimates from the Congo swamp forests. CO_2_‐equivalent emissions in the Zou et al. (2022) dataset were calculated using Equation (1):

(1) $${\mathrm{CO}}_{2}\text{equivalent}={\mathrm{CO}}_{2}+34{\mathrm{CH}}_{4}+298N_{2}O$$

where CO_2_ represents net ecosystem exchange of vertical fluxes and the conversion factors of 34 and 298 for CH_4_ and N_2_O are global warming potential relative to CO_2_ over a 100‐year horizon (Zou et al. 2022), consistent with IPCC reporting conventions (IPCC 2013). To assess the shorter‐term implications of GHG flux changes, we also calculated CO_2_‐equivalent emissions using global warming potentials over a 20‐year horizon. Here, weights of 82.5 and 273 were used for CH_4_ and N_2_O (IPCC 2021; Figure S2). Flood extent pixels for each year were grouped into the following WTL classes: ≤ −70 cm, −70 to −50 cm, −50 to −30 cm, −30 to −5 cm, −5 to 40 cm and > 40 cm. The GHG flux estimates per unit area were then applied to each WTL class to provide total swamp forest emissions per year for CO_2_, CH_4_, N_2_O and CO_2_‐equivalent (Figure 3 and Table S4).

## Author Contributions

A.B. designed and performed the analyses and prepared the first manuscript draft; J.W. contributed to the project design and revised the manuscript draft; N.P. contributed to the project design and revised the manuscript draft.

## Funding

This work was supported by the Department for Environment, Food and Rural Affairs, UK Government; Research England.

## Conflicts of Interest

The authors declare no conflicts of interest.

## Supporting information


**Data S1:** gcb70746‐sup‐0001‐Supinfo.pdf.

## Data Availability

ALOS PALSAR and PALSAR‐2 imagery, Climate Hazards Group InfraRed Precipitation with Station data (CHIRPS), tropical moist forest landcover, MODIS EVI and MERIT Hydro data can be accessed through Google Earth Engine (https://earthengine.google.com/). Satellite altimeter‐derived river level height data can be obtained from NASA (https://blueice.gsfc.nasa.gov/gwm). The ground‐based GPS coordinates for Congo peat swamps are available from Crezee et al. (2022) and tropical wetland GHG flux data are available from Zou et al. (2022). Both pre‐processed datasets used in this study, as well as all python code used for the analyses, are available via Zenodo at https://doi.org/10.5281/zenodo.15623477 (Byrne 2025).
