# Supplementary material for: Wetland Expansion Reduces CO2 ‐Equivalent Emissions and Strengthens the Congo Basin's Role as a Net Carbon Sink
Source: Glob Chang Biol. 2026 Feb 12;32(2):e70746. doi: 10.1111/gcb.70746 (PMC12895296; doi:10.1111/gcb.70746)
Supplement: Supplementary file 1 — Data S1: gcb70746‐sup‐0001‐Supinfo.pdf. [file GCB-32-e70746-s001.pdf]

# Supporting Information

**Validation of swamp forest extents and hydrological trends**

Previous estimates of wetland extent in the Congo Basin vary depending on the methods used and the ecosystems under investigation, from 167,600 $km^2$ for permanent peat swamps (Crezee et al., 2022) to 359,556 $km^2$ for all wetlands (Bwangoy et al., 2010). Our swamp forest extent estimates, including both peat-forming and non-peat-forming swamp forests experiencing year-round inundation, fall within this range, providing confidence in our results. The GIEMS-2 dataset also observed increasing wetland areas up to 2020 (340 $km^2$/year, P = 0.068), however, data were not available to compare trends up to 2024. GIEMS-2 wetland extent estimates were substantially lower than our PALSAR swamp forest extent estimates. This difference stems from fundamental differences in detection capabilities, specifically GIEMS-2 relying on microwave emissivity at 37 GHz which is strongly attenuated by dense tropical forest canopies, leading to the underestimation of inundated forests (Prigent et al., 2020). Its coarse spatial resolution (25km by 25km) further prevents detection of the narrow channels and vegetated floodplains that dominate the Cuvette Centrale. Regardless, the observed increase in wetland extents provides further support for a basin-wide wetting trend. Our swamp forest extent trends between 2007 and 2024 were also compared to precipitation and river water level datasets for further validation. Basin-wide annual average rainfall derived from Climate Hazards Group InfraRed Precipitation with Station data (CHIRPS) showed a non-significant increasing trend between 2007 and 2024 (1.39 mm/year, P = 0.625). However, annual rainfall in 2024 was considerably lower than previous years and the rainfall trend showed a significant increase up to 2023 (4.93 mm/year, P = 0.036; Fig. 1d). Similarly, annual average river level height anomalies for 52 river stations across the basin increased over the study period (0.02 m/year, P = 0.051) (Fig. 1d). Both rainfall and river level height anomalies declined between 2007 and 2014, in line with the previously described drying trend (Jiang et al., 2019), before returning to a wetter period up to 2023. The decline in rainfall and river level heights in 2024 was not observed in swamp forest extent, likely due to the lag between climatic variation and wetland hydrology (Ivory et al., 2019).

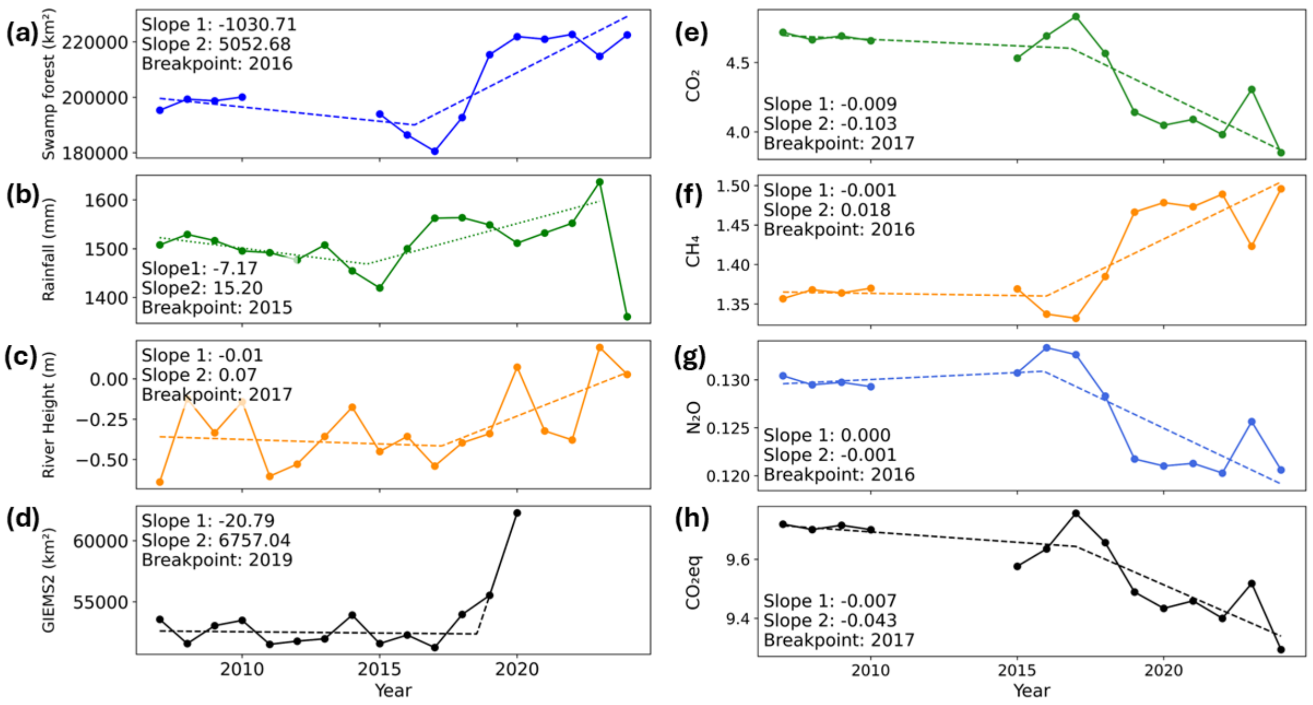


**Figure S1. Breakpoint analyses of hydrological and greenhouse gas (GHG) flux trends.** Comparison between timeseries shifts and trend slopes in hydrological trends for a) swamp forest extent ($km^2$), b) annual average basin-wide rainfall (mm), c) river level height (m), and d) GIEMS-2 wetland extents ($km^2$) (related to Figure 1). Rainfall in 2024 was anomalously low and was excluded from the analysis. Comparison between timeseries shifts and trend slopes in annual GHG fluxes for e) $CO_2$ (100 million tonnes), f) $CH_4$ (10 million tonnes), g) $N_2O$ (million tonnes), and h) $CO_2$-equivalant (100 million tonnes) (related to Figure 3).

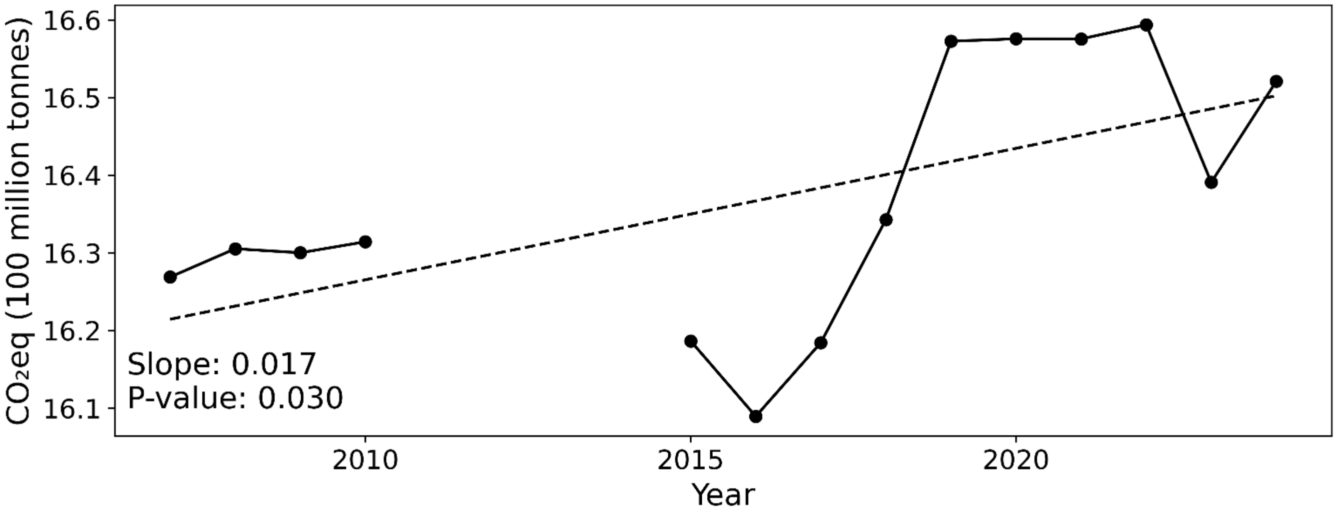


**Figure S2. Annual swamp forest $CO_2$-equivalent emissions based on global warming potential on a 20-year horizon.** Conversion factors for $CO_2$-equivalent emissions assume weights of 82.5 for $CH_4$ and 273 for $N_2O$. Units are 100 million tonnes of $CO_2$-equivalent emissions. As shown here, considering a 20-year horizon for global warming potential leads to the opposite trend in $CO_2$-equivalent emissions than when considering a 100-year horizon i.e. the IPCC reporting standard. This shift is underpinned by the increased strength in methane's global warming potential over shorter timeframes. Related to Figure 3.

**Table S1. Annual swamp forest extent in $km^2$ for the Congo River Basin.** Confidence intervals were quantified using the random forest classification accuracy assessment.

| Year | Swamp forest extent ($km^2$) | Lower 95 % CI ($km^2$) | Upper 95 % CI ($km^2$) |
|---|---|---|---|
| 2007 | 195,344.76 | 184,742.00 | 205,947.52 |
| 2008 | 199,324.93 | 188,506.14 | 210,143.73 |
| 2009 | 198,740.81 | 187,953.72 | 209,527.90 |
| 2010 | 200,110.58 | 189,249.14 | 210,972.02 |
| 2015 | 193,969.55 | 183,441.43 | 204,497.67 |
| 2016 | 186,479.51 | 176,357.93 | 196,601.09 |
| 2017 | 180,592.08 | 170,790.05 | 190,394.11 |
| 2018 | 192,751.06 | 182,289.08 | 203,213.05 |
| 2019 | 215,384.61 | 203,694.14 | 227,075.08 |
| 2020 | 221,889.86 | 209,846.30 | 233,933.41 |
| 2021 | 220,937.78 | 208,945.90 | 232,929.66 |
| 2022 | 222,685.54 | 210,598.79 | 234,772.28 |
| 2023 | 214,820.45 | 203,160.60 | 226,480.30 |
| 2024 | 222,467.40 | 210,392.50 | 234,542.30 |

**Table S2. Swamp forest extent trends for the nine subbasins within the Congo River Basin.** Mean swamp forest extent is for the 2007 to 2024 study period. The slope represents the change per year ($km^2$) in swamp forest extent for each subbasin. The HYBAS ID represents the unique subbasin identifier in the HydroSHEDS Basins Level 4 dataset (Linke et al., 2019).

| HYBAS ID (HydroSHEDS Basins Level 4) | Subbasin name | Subbasin size ($km^2$) | Mean swamp forest extent ($km^2$) | Slope (swamp forest change in $km^2$ per year) | Slope CIs | P value |
|---|---|---|---|---|---|---|
| 1041156950 | Ubangui | 648,276.2 | 29,735.73 | 178.88 | 10.41 – 347.36 | 0.04 |
| 1041213640 | Kasai | 894.488.8 | 28,373.13 | 210.85 | 25.66 – 396.04 | 0.03 |
| 1041156960 | Middle Congo | 973,777.8 | 94,882.20 | 645.57 | -214.58 – 1,505.72 | 0.13 |
| 1041174950 | Sangha | 285,042.7 | 43,358.36 | 420.21 | 294.54 – 545.87 | <0.001 |
| 1041174960 | Tumba | 14,082.8 | 3,817.26 | 27.74 | 14.53 – 40.95 | <0.001 |
| 1041213630 | Lower Congo | 67,657.6 | 4,240.61 | 43.62 | 18.96 – 68.28 | 0.002 |
| 1040020040 | Kinshasa | 102,307.3 | 269.32 | 1.69 | -4.58 – 7.95 | 0.57 |
| 1041259940 | Upper Congo | 453,077.6 | 1.84 | -0.03 | -0.11 – 0.05 | 0.47 |
| 1041259950 | Tanganyika | 266,510.8 | 0.00 | NA | NA | NA |

**Table S3. Greenhouse gas (GHG) fluxes in tonnes per km$^2$ for tropical wetlands.** Data were obtained from a synthesis of 504 global GHG net flux experimental studies conducted by Zou *et al.* (2022) and filtered to contain only tropical wetlands. All emissions estimates and 95 % confidence intervals are converted to tonnes per km$^2$ for consistency.

| Water table level (WTL) (cm) | $CO_2$ (95 % CI) | $CH_4$ (95 % CI) | $N_2O$ (95 % CI) | $CO_2$-equivalent (95 % CI) |
|---|---|---|---|---|
| ≤-70 | 5,861 (2206 – 9516) | 0.378 (-0.49 – 1.246) | 0.827 (0.27 – 1.383) | 6,120 (2270 – 9970) |
| -70 to -50 | 740 (-549 – 2028) | 1.042 (-0.056 – 2.141) | 1.784 (-0.272 – 3.84) | 1,307 (-632 – 3245) |
| -50 to -30 | 2,099 (1677 – 2521) | 4.131 (0.34 – 7.921) | 0.957 (0.052 – 1.862) | 2,525 (1704 – 3345) |
| -30 to -5 | -268 (-897 – 360) | 33.478 (12.973 – 53.983) | 0.739 (0.366 – 1.111) | 1,090 (-347 – 2527) |
| -5 to 40 | -1,400 (-1976 – -824) | 60.298 (30.456 – 90.14) | 0.803 (0.473 – 1.133) | 889 (-800 – 2578) |
| >40 | 332 (-331 – 994) | 91.171 (56.044 – 126.298) | 0.198 (0.126 – 0.269) | 3,490 (1612 – 5368) |

**Table S4. Annual Congo wetland GHG emissions (tonnes) estimated from swamp forest extents and water table levels.**

| Year | $CO_2$ (95% CI) | $CH_4$ (95% CI) | $N_2O$ (95% CI) | $CO_2$-equivalent (100-yr) (95% CI) |
|---|---|---|---|---|
| 2007 | 471,872,289 (112,750,941 - 830,862,232) | 13,568,742 (8,015,462 - 19,122,023) | 130,422 (54,363 - 206,273) | 971,934,450 (356,736,226 - 1,587,126,319) |
| 2008 | 466,461,853 (110,107,816 - 822,683,431) | 13,681,641 (8,086,520 - 19,276,762) | 129,475 (54,273 - 204,468) | 970,079,492 (356,585,742 - 1,583,567,143) |
| 2009 | 469,243,111 (111,333,131 - 827,021,018) | 13,640,738 (8,061,668 - 19,219,808) | 129,738 (54,304 - 204,963) | 971,548,647 (357,084,597 - 1,586,006,590) |
| 2010 | 465,841,991 (109,775,289 - 821,775,857) | 13,700,654 (8,099,607 - 19,301,701) | 129,289 (54,165 - 204,205) | 970,050,619 (356,896,104 - 1,583,198,996) |
| 2015 | 453,239,895 (105,005,096 - 801,345,887) | 13,693,769 (8,067,031 - 19,320,508) | 130,726 (55,892 - 205,356) | 957,643,482 (346,330,825 - 1,568,950,708) |
| 2016 | 469,229,267 (112,561,454 - 825,772,382) | 13,373,574 (7,861,656 - 18,885,491) | 133,351 (56,670 - 209,828) | 963,530,276 (346,038,705 - 1,581,016,419) |
| 2017 | 483,341,872 (117,780,848 - 848,777,730) | 13,319,973 (7,843,615 - 18,796,332) | 132,626 (56,302 - 208,744) | 975,603,966 (353,189,188 - 1,598,013,936) |
| 2018 | 456,691,308 (105,244,109 - 808,006,101) | 13,849,553 (8,184,633 - 19,514,474) | 128,309 (54,830 - 201,581) | 965,669,502 (353,895,767 - 1,577,438,196) |
| 2019 | 414,160,021 (85,641,271 - 742,534,596) | 14,665,194 (8,709,695 - 20,620,693) | 121,743 (52,363 - 190,911) | 948,908,194 (354,665,255 - 1,543,145,359) |
| 2020 | 404,778,922 (82,010,786 - 727,399,918) | 14,785,053 (8,787,292 - 20,782,814) | 121,018 (51,526 - 190,299) | 943,385,800 (354,130,591 - 1,532,634,239) |
| 2021 | 408,995,541 (83,788,249 - 734,056,257) | 14,732,886 (8,756,188 - 20,709,583) | 121,290 (51,585 - 190,784) | 945,910,067 (355,101,931 - 1,536,711,504) |
| 2022 | 398,010,927 (78,947,531 - 716,926,385) | 14,891,533 (8,851,515 - 20,931,551) | 120,282 (51,552 - 188,800) | 940,018,131 (352,857,818 - 1,527,171,891) |
| 2023 | 430,648,799 (94,830,513 - 766,325,699) | 14,232,063 (8,435,344 - 20,028,782) | 125,644 (52,309 - 198,769) | 951,836,243 (353,971,603 - 1,549,693,231) |
| 2024 | 384,985,260 (74,141,663 - 695,680,671) | 14,960,103 (8,881,198 - 21,039,008) | 120,612 (51,810 - 189,206) | 929,422,505 (346,872,289 - 1,511,965,759) |

# References

Bwangoy, J. R. B., Hansen, M. C., Roy, D. P., Grandi, G. De, & Justice, C. O. (2010). Wetland mapping in the Congo Basin using optical and radar remotely sensed data and derived topographical indices. *Remote Sensing of Environment*, *114*(1), 73–86. https://doi.org/10.1016/j.rse.2009.08.004

Crezee, B., Dargie, G. C., Ewango, C. E. N., Mitchard, E. T. A., Emba B, O., Kanyama T, J., Bola, P., Ndjango, J. B. N., Girkin, N. T., Bocko, Y. E., Ifo, S. A., Hubau, W., Seidensticker, D., Batumike, R., Imani, G., Cuní-Sanchez, A., Kiahtipes, C. A., Lebamba, J., Wotzka, H. P., … Lewis, S. L. (2022). Mapping peat thickness and carbon stocks of the central Congo Basin using field data. *Nature Geoscience*, *15*(8), 639–644. https://doi.org/10.1038/s41561-022-00966-7

Ivory, S. J., McGlue, M. M., Spera, S., Silva, A., & Bergier, I. (2019). Vegetation, rainfall, and pulsing hydrology in the Pantanal, the world's largest tropical wetland. *Environmental Research Letters*, *14*(12). https://doi.org/10.1088/1748-9326/ab4ffe

Jiang, Y., Zhou, L., Tucker, C. J., Raghavendra, A., Hua, W., Liu, Y. Y., & Joiner, J. (2019). Widespread increase of boreal summer dry season length over the Congo rainforest. In *Nature Climate Change* (Vol. 9, Number 8, pp. 617–622). Nature Publishing Group. https://doi.org/10.1038/s41558-019-0512-y

Linke, S., Lehner, B., Ouellet Dallaire, C., Ariwi, J., Grill, G., Anand, M., Beames, P., Burchard-Levine, V., Maxwell, S., Moidu, H., Tan, F., & Thieme, M. (2019). Global hydro-environmental sub-basin and river reach characteristics at high spatial resolution. *Scientific Data*, *6*(1). https://doi.org/10.1038/s41597-019-0300-6

Prigent, C., Jimenez, C., & Bousquet, P. (2020). Satellite-Derived Global Surface Water Extent and Dynamics Over the Last 25 Years (GIEMS-2). *Journal of Geophysical Research: Atmospheres*, *125*(3). https://doi.org/10.1029/2019JD030711

Zou, J., Ziegler, A. D., Chen, D., McNicol, G., Ciais, P., Jiang, X., Zheng, C., Wu, Jie, Wu, Jin, Lin, Z., He, X., Brown, L. E., Holden, J., Zhang, Z., Ramchunder, S. J., Chen, A., & Zeng, Z. (2022). Rewetting global wetlands effectively reduces major greenhouse gas emissions. *Nature Geoscience*, *15*(8), 627–632. https://doi.org/10.1038/s41561-022-00989-0
